# Environmental Conditions around Itineraries to Destinations as Correlates of Walking for Transportation among Adults: The RECORD Cohort Study

**DOI:** 10.1371/journal.pone.0088929

**Published:** 2014-05-14

**Authors:** Noëlla Karusisi, Frédérique Thomas, Julie Méline, Ruben Brondeel, Basile Chaix

**Affiliations:** 1 Inserm, U707, Paris, France; 2 Université Pierre et Marie Curie-Paris6, UMR-S 707, Paris, France; 3 Centre d'Investigations Préventives et Cliniques, Paris, France; Arizona State University, United States of America

## Abstract

**Introduction:**

Assessing the contextual factors that influence walking for transportation is important to develop more walkable environments and promote physical activity. To advance previous research focused on residential environments and overall walking for transportation, the present study investigates objective environmental factors assessed around the residence, the workplace, the home – work itinerary, and the home – supermarket itinerary, and considered overall walking for transportation but also walking to work and to shops.

**Methods:**

Data from the RECORD Study involving 7290 participants recruited in 2007–2008, aged 30–79 years, and residing in the Paris metropolitan area were analyzed. Multilevel ordinal regression analyses were conducted to investigate environmental characteristics associated with self-reported overall walking for transportation, walking to work, and walking to shops.

**Results:**

High individual education was associated with overall walking for transportation, with walking to work, and walking to shops. Among workers, a high residential neighborhood education was associated with increased overall walking for transportation, while a high workplace neighborhood education was related to an increased time spent walking to work. The residential density of destinations was positively associated with overall walking for transportation, with walking to work, and with walking to shops, while the workplace density of destinations was positively associated with overall walking for transportation among workers. Environmental factors assessed around the itineraries were not associated with walking to work or to the shops.

**Conclusion:**

This research improves our understanding of the role of the environments on walking for transportation by accounting for some of the environments visited beyond the residential neighborhood. It shows that workers' walking habits are more influenced by the density of destinations around the workplace than around the residence. These results provide insight for the development of policies and programs to encourage population level active commuting.

## Introduction

Regular physical activity helps to increase quality of life [1], [2] and is associated with a reduced risk of cardiovascular diseases, cancer, and other chronic diseases [3], [4]. Besides leisure time sport activity [5], [6], walking is one of the most common and popular forms of physical activity. Its appeals for health promotion are related to the fact that it is accessible to all, requires little skill, and is associated with a low risk of injury. Several studies have demonstrated the positive health effects of overall walking (recreational and for transportation) [7], [8], [9] and numerous studies have focused on the environmental determinants of walking [10], [11], [12]. Walking for transportation, defined as walking to engage in activities at the trip end (e.g., working, shopping) [13], [14], may be key to meeting recommended levels of physical activity. This potential is reflected in the US Healthy People 2020 objective PA-13.1, which calls adults to walk more frequently for transportation [15].

Most of the environmental research related to walking has focused on the built environment [16], [17], [18]. As the part of the physical environment that is constructed by human activity, the built environment comprises: land use patterns, the distribution across space of activities and the buildings that house them; the transportation system, the physical infrastructure of roads, sidewalks, bike paths, etc., as well as the services that this system provides; and urban design as the arrangement and appearance of the physical elements in a community [19].

A large number of studies have reported positive relationships between the spatial accessibility to shops/services/work and walking for transportation [20], [21]. However, as with other health outcomes [22], [23], [24], most of the studies that investigated the relationships between the built environment and transportation walking have exclusively focused on the residential environment, which is a severe limitation because people do not only walk around their residence (e.g., many people may walk more around their workplace than around their home). To our knowledge, only few studies have accounted for non-residential environments [25], [26], [27]. To address this gap, the present study on walking for transportation investigates not only the residential environment but also the workplace environment, the environment around the residence–workplace itinerary, and the environment around the residence–supermarket itinerary, in relation to specifically related outcomes (walking to work and walking for shopping). While it is important to focus on the overall physical activity level of people, it is also useful to investigate specific and well-defined physical activity outcomes to identify their individual/environmental determinants for which efficient interventions may be easier to determine. The workplace and supermarket destinations were considered because, according to the 2010 Global Survey on Transportation for the Ile-de-France region, the most frequently visited locations were the workplace and the supermarkets [28].

Overall, the main purpose of the present study was to examine the correlates of overall walking and walking to certain destinations and to account for the environments the participants were exposed to during these specific walking episodes, in order to assess which environments (at the start of the trip, at the destination, or along the trip) and which environmental characteristics were more likely to influence walking for transportation.

## Material and Methods

### Ethics Statement

The study protocol was approved by the French Data Protection Authority. All the participants signed an informed consent to enter the study.

### Study population

Data from the RECORD Cohort Study (“Residential Environment and CORonary heart Disease”, www.record-study.org) were used for the analyses [29]. As described elsewhere, 7290 participants were recruited between March 2007 and February 2008 [15], [29], [30], [31], [32], [33]. The participants benefitted from a free medical checkup offered every 5 years by the French National Health Insurance System for Salaried Workers to all working and retired employees and their families. Participants were recruited without a priori sampling during these 2-hour–long preventive checkups conducted by the Centre d'Investigations Préventives et Cliniques in 4 of its health centers, located in the Paris metropolitan area (Paris, Argenteuil, Trappes, and Mantes-la-Jolie). Eligibility criteria were as follows: age 30 to 79 years; ability to complete study questionnaires; and residence in one of the 10 (out of 20) administrative divisions of Paris or 111 other municipalities of the metropolitan area selected a priori. Of the persons invited for participation, 83.6% accepted to participate and completed the data collection protocol. All participants underwent a physical examination, completed questionnaires, and were geocoded based on their residential address in 2007–2008. Research assistants rectified all incorrect or incomplete addresses with the participants by telephone. Extensive investigations with local Departments of Urbanism were conducted to complete the geocoding. Precise spatial coordinates were identified for 100% of the participants. The study protocol was approved by the French Data Protection Authority. After excluding individuals with missing values for walking, there were 7105 participants from 1908 census block group neighborhoods (IRIS neighborhoods, i.e., local geographic subdivisions of municipalities) in the database. Municipalities were used as a selection criterion for the recruitment of participants, while the more local IRIS neighborhoods were considered for the multilevel analyses.

### Assessment of participants' workplaces

Administrative files from CNAV (National Old Age Insurance System) and Insee (National Institute of Statistics and Economic Studies) were used to assess and geocode participants' workplaces. First, we identified the establishment of work based on the CNAV database of occupational careers that is used for the computation of retirement pensions. The file received from CNAV indicated the employer (or employers, with a maximum of 3) of each participant for each year, with the corresponding establishment identification code. The file did not provide information on the dates of beginning and end of the contracts during the year. The data therefore did not allow us to confirm that the participant was employed, nor with which employer (if several employers were reported), at the exact date of enrollment in the study. We retained for every year only the main employer, which was the one from which the participant received the most important salary. To be sure to only consider workplaces where the participant was already working (or had worked) at the time of recruitment in the study (and thus avoid reverse causality problems), we assigned to each individual the main work establishment of the year preceding his/her inclusion in the study.

We then used databases of facilities or companies from Insee (Permanent Database of Facilities, SIRENE register) or from Trade Dimension to geocode the workplaces. These databases allowed us to retrieve the spatial coordinates of the workplace of the participants. For the 4536 participants (of the 7290 of the RECORD Cohort) for whom a work establishment was identified, we were able to retrieve in these databases the coordinates of the workplace of 3837 participants (geocoding at the workplace address). For 254 other participants, the workplace was geocoded using Google Maps based on addresses found in company directories available on the internet. The workplace of respectively 238 and 123 participants was geocoded at the centroid of the corresponding census block group neighborhood or at the centroid of the corresponding municipality. Eighty-four participants for whom the workplace could not be identified or located and 124 participants for whom the workplace was located outside the Paris Ile-de-France region were excluded from the analyses. Overall, a workplace was geocoded for 4331 participants (of 7290) residing and working in the Ile-de-France region.

After excluding individuals with missing values for walking and participants who were inactive or had no geocoded workplace, there were 4127 participants from 1666 census block group neighborhoods (IRIS neighborhoods) in the database.

### Assessment of participants' supermarkets

The participants were asked to report the brand and address of the supermarket where they did most of their food shopping [28], [34]. However, each participant's primary supermarket could not always be straightforwardly identified. Also, in certain cases, there was more than one supermarket of the same brand in the same street. In these cases and others, participants were systematically telephoned in the months subsequent to their health examination, in attempts to precisely identify the supermarket where they shopped. Technicians conducting these calls used Google Maps and the websites of the different supermarket brands to assist them in their searches. In the end, the official business identification code (SIRET) of each supermarket was retrieved. The supermarkets were geocoded through the linkage of exact geographic coordinates from databases of Insee and Trade Dimension, or through manual geocoding.

Among the 7290 participants, 7131 participants were coded as conducting most of their food shopping in 1097 distinct supermarkets after exclusion of participants who reported doing most of their shopping at the market, participants who did most of their food shopping via the internet, and those who did not make most of their shopping in a given supermarket or could not provide enough information to identify the supermarket.

After excluding individuals with missing values for walking and those who had no geocoded primary supermarket, there were 6958 participants from 1905 census tracts (IRIS neighborhoods) in the database.

### Measures

#### Outcome measures

Data related to walking for transport were obtained from the baseline questionnaire. Participants were asked to report the number of hours and minutes they had walked over the previous 7 days, separately for commuting to work, for shopping, and for going to other destinations.

Four complementary outcomes were created: (i) overall walking time for transport (by summing time in the 3 walking categories listed above) (defined in the whole population, sample size for analysis  = 7105); (ii) overall walking time for transport among workers (n = 4127); (iii) walking time for home–work commuting among workers (n = 4127); and (iv) overall walking time to shops among participants for whom a supermarket was geocoded (n = 6958). Five-category ordinal variables were created for the outcomes, with the first category corresponding to 0 minute walked over the previous 7 days, the second category corresponding to 0 to 15 minutes walked per day over the previous 7 days, the third category corresponding to 15 to 30 minutes walked per day over the previous 7 days, the fourth category corresponding to 30 to 60 minutes, and the fifth category corresponding to over 60 minutes walked per day over the previous 7 days.

It is relevant to analyze the overall walking time for transport among workers (not only in the total sample) due to the specificity of this population. First, workers may have busier schedules than other participants. Second, workers may have the opportunity to walk around their workplace if there are opportunities available in the geographic environment.

#### Individual adjustment measures

Several socio-demographic characteristics were considered: age, sex, individual education, marital status, occupation, household income, homeownership, financial strain, and Human Development Index of each participant's country of birth. Age was divided in 3 classes (30–44, 45–59, and 60 years or older). Education was divided in 4 classes: no education; primary education and lower secondary education; higher secondary education and lower tertiary education; and upper tertiary education. Marital status was coded in 2 classes: living alone or as a couple. Occupation was coded in 4 categories: high white-collar workers, intermediate occupations, low white-collar workers, and blue-collar workers. Ownership of dwelling was coded as a binary variable. Household income adjusted for household size was divided into 4 categories. We attributed to each individual the 2004 Human Development Index (HDI) of his/her country of birth [35], as a crude proxy of his/her cultural origin. Following the United Nations Development Program, a categorical variable was used to distinguish people born in low-development countries (HDI<0.5), in medium-development countries (HDI between 0.5 and 0.8), in France, and in other high-development countries (HDI>0.8).

#### Distance to destinations

The ArcInfo 10 Software (ESRI 2011. ArcGIS Desktop: Release 10. Redlands, CA: Environmental Systems Research Institute) and its Network Analyst, applied to street network data from the National Geographic Institute, were used to estimate the shortest street network distance between each respondent's home and their workplace and the shortest distance between each participant's residence and their primary supermarket. Such shortest routes were also used for the calculation of environmental exposures along the home – work or home – supermarket itineraries.

#### Neighborhood measures

Regarding the socio-demographic environment, we considered neighborhood education (proportion of residents aged >15 years with an upper tertiary education from the 2006 census). Regarding the service environment, we considered the density of destinations (number of services accessible in the neighborhood, Permanent Database of Facilities of Insee). The proportion of the area covered with parks or green spaces and the connected node ratio (number of street intersections with at least 3 ways divided by the number of intersections plus cul-de-sacs) were also considered as physical environment factors.

**Figure 1:**
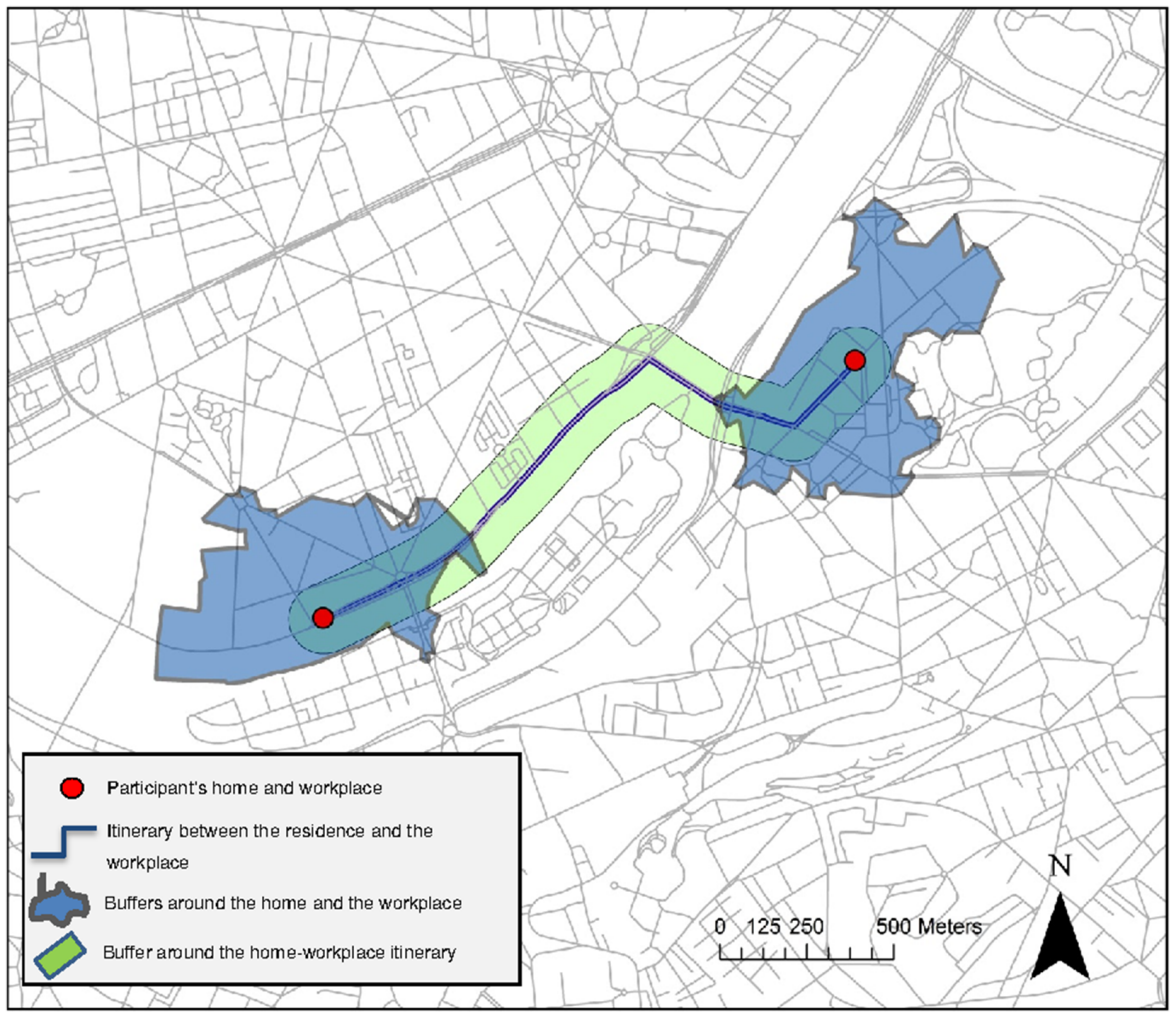
Assessment of the environmental characteristics around the residence, the destinations, and the itineraries between them.

As illustrated in Figure 1, all these environmental factors were determined within street-network based residential neighborhoods, within street-network based workplace neighborhoods, in a buffer along the street network itinerary between the residence and the workplace, and in a buffer along the street network itinerary between the residence and the primary supermarket. The buffers around the residence and the workplace had a street network radius of 500 m (as a walkable area around these two major anchor points in individuals' lives). The buffers around the itineraries had a Euclidean radius of 100 m (a shorter radius was used because itineraries are not considered to be anchor points but transitory locations). All the contextual variables were determined with Python scripts based on ArcInfo 10. The neighborhood variables were not determined around the supermarket because, contrary to the workplace, the supermarket is not a major spatial anchor point from which people access to services (especially because people typically go at the supermarket and then have to go back home with the items that they purchased).

### Statistical analysis

To analyze associations between individual and environmental variables and walking for transportation, we estimated several multilevel logit ordinal regression models using the Dual Quasi-Newton optimization estimation approach of Proc Glimmix in SAS 9.3 (SAS Institute Inc., Cary, North Carolina). The coefficients estimated were used to calculate the odds ratio for being in a given category of the outcome variable rather than in the immediately adjacent category. The random effect of the models allowed us to account for the within-neighborhood correlation in each outcome, with participants nested within IRIS administrative neighborhoods (the median number of residents in 2006 in these neighborhoods was 2536, interquartile range: 2161, 3115) [16]. The model building involved several steps:

1. We included all the individual socio-demographic variables.
2. We then added the distance to the workplace to the model estimated among workers for walking to work; and we added the distance to the primary supermarket to the model for walking to shops (distances to work and to the main supermarket were not correlated with overall walking as our aim was to estimate as specific relationships as possible).
3. We then tested one by one the relevant environmental characteristics (related to the residential and workplace environments, itinerary between the residence and the workplace, and itinerary between the residence and supermarket), in models adjusted for individual covariates and distances to work/supermarket (when relevant). Each environmental variable was tested in a separate model.
4. Finally, we progressively combined into one model the variable on the distance to destination (when relevant) and the contextual variables that were independently associated with each outcome (individual adjustment variables were systematically retained in the model, while distance and environmental variables were included only if they were associated).

## Results

Overall, the median time spent walking to work, to shops, and to other places over the previous 7 days was 240 minutes (interquartile range  = 120 minutes; 450 minutes). Among workers, the median time spent walking to work, to shops, or to other places over the previous 7 days was 240 minutes (interquartile range  = 120 minutes; 430 minutes). The median time spent walking to work among workers over the previous 7 days was 75 minutes (interquartile range  = 0 minutes; 180 minutes). Finally, the median time spent walking to shops was 90 minutes (interquartile range  = 30 minutes; 180 minutes). The median distance to work was 8178 m (interquartile range: 4362, 13927 m) while the median distance to one's main supermarket was 1029 m (interquartile range: 430, 2515 m). The median number of services available in 500 m buffers around the residence and workplace was, respectively, 371 (interquartile range: 96, 1150) and 149 (interquartile range: 35, 496). Descriptive information on the different subsamples is provided in Table 1.

**Table 1:** Descriptive information on the subsamples used in the study based on the RECORD Cohort, Paris Metropolitan Area, 2007–2008.

Variables	Total sample (n = 7105)	Working population (n = 4127)	Population with a supermarket (n = 6958)
Sex			
Men	65.6%	71.7%	65.5%
Women	34.4%	28.3%	34.6%
Age (years)			
30–44	35.5%	44.7%	35.5%
45–59	41.7%	46.4%	41.7%
60–79	22.9%	8.9%	22.8%
Individual education			
No education	7.5%	7.2%	7.5%
Medium-low education	24.1%	21.1%	24.3%
Medium-high education	29.5%	29.1%	29.6%
High education	38.1%	41.7%	37.9%
Marital status			
Living alone	29.8%	28.8%	29.8%
Living as a couple	64.7%	65.3%	64.7%
Occupation			
High white-collar workers	60.3%	45.8%	39.4%
Intermediate occupations	5.5%	6.9%	5.6%
Low white-collar workers	38.2%	33.1%	38.5%
Blue-collar workers	11.0%	14.3%	11.0%
Homeownership			
Owners	54.5%	52.2%	54.4%
Non owners	45.4%	47.6%	45.5%
Household income			
Low income	25.5%	25.3%	25.5%
Medium-low income	24.2%	24.3%	24.3%
Medium-high income	22.7%	23.3%	22.7%
High income	27.2%	26.6%	26.9%
Perceived financial strain	16.7%	17.4%	16.7%
Human Development Index of country of birth			
Low	4.9%	5.8%	4.9%
Medium	15.4%	15.8%	15.4%
France	70.5%	68.5%	70.4%
High (other than France)	9.2%	9.9%	9.2%

### Associations between the environmental variables and overall walking for transportation

Associations between individual factors and overall walking for transportation are shown in Table S1, while associations between environmental factors not adjusted for each other and overall walking for transportation are shown in Table S2.

After controlling for all individual characteristics, when we combined the different contextual variables into one model, only the density of destinations around the residence remained associated, strongly and with a dose-response pattern, with overall walking for transportation in the whole sample (Table 2). Regarding the same outcome among workers, a mid-high or high residential neighborhood education and a high density of destinations around the workplace (not around the residence as in the whole sample) remained independently associated with a higher overall walking time for transportation after mutual adjustment (Table 2).

**Table 2:** Associations between environmental characteristics and walking for transportation, the RECORD Study, 2007–2008*.

Variables	Overall walking for transportation (n = 7105)	Overall walking for transportation among workers (n = 4127)
	OR (95% CI)	OR (95% CI)
Residential neighborhood education (vs. low)		
Mid-low	–	1.08 (0.92–1.28)
Mid-high	–	1.36 (1.15–1.62)
High	–	1.26 (1.05–1.50)
Density of destinations around the residence (vs. low)		
Mid-low	1.31 (1.16–1.47)	–
Mid-high	1.64 (1.46–1.86)	–
High	2.20 (1.94–2.49)	–
Density of destinations around the workplace (vs. low)		
Mid-low	–	1.05 (0.89–1.22)
Mid-high	–	1.22 (1.04–1.42)
High	–	1.39 (1.19–1.63)

*Models adjusted for age, sex, marital status, individual education, occupation, home ownership status, perceived financial strain, household income, and the level of human development of the country of birth.

### Associations between the environmental variables and walking to work or to shops

Associations between individual factors and walking to work or shops are shown in Table S3, while associations between distance to destinations or environmental factors not adjusted for each other and walking to work or shops are shown, respectively, Tables S4 and S5.

After controlling for individual socioeconomic characteristics, when the different environmental variables associated in separate models were combined into one model, only a high residential density of destinations and a high workplace neighborhood education remained associated with walking to work (Table 3). Regarding walking to shops, only a high density of destination around the residence was associated with the outcome after controlling for individual socioeconomic characteristics (only the former association showed a dose-response pattern) (Table 3). Neither the distance to work nor the distance to the supermarket was associated with walking to work or shops after adjustment (although a short distance to the supermarket was associated with increased walking to shops before such adjustment).

**Table 3:** Associations between environmental characteristics and walking to work and to shops, the RECORD Study, 2007–2008*.

Variables	Walking to work (n = 4127)	Walking to shops (n = 6958)
	OR (95% CI)	OR (95% CI)
Density of destinations around the residence (vs. low)		
Mid-low	1.19 (1.01–1.40)	1.17 (1.04–1.33)
Mid-high	1.50 (1.26–1.77)	1.53 (1.35–1.74)
High	1.66 (1.37–2.01)	1.69 (1.48–1.91)
Workplace neighborhood education (vs. low)		
Mid-low	1.14 (0.97–1.35)	–
Mid-high	0.95 (0.79–1.13)	–
High	1.21 (1.00–1.47)	–

*These models included all individual sociodemographic variables, and the environmental variables associated with the outcome.

## Discussion

### Study findings

The environmental factors tested in this study were chosen based on hypotheses of effects on walking: street network connectivity as enabling walking, destinations as motivating walking, and socioeconomic status and green spaces as contributing to the agreeableness of the environment. Independent associations were documented for different walking outcomes with neighborhood socioeconomic status and the density of destinations.

Among workers, a high residential neighborhood education was associated with overall walking for transportation while a high workplace neighborhood education was positively related to walking to work. Neighborhood socioeconomic status has often been found to be associated with active transportation [36], [37] and with the level of physical activity [38], [39]. The fact that a high residential neighborhood education was associated with overall walking for transportation is coherent with our hypothesis that a high socioeconomic status in the neighborhood creates an agreeable atmosphere that encourages walking to a number of proximate destinations. The relatively weak relationship that was documented between workplace neighborhood education and walking to work is of interest, as it suggests that, beyond the residential neighborhood, attributes of destinations also have their importance.

The most consistent finding was the strong relationships documented with the density of services and destinations [12]. This result is consistent with previous research on the effects of the built environment on physical activity in general and walking for transportation in particular [40], [41], [42]. Our results confirm findings from previous studies that used the walkability index to capture the spatial access to walking destinations [14], [43]. In our study, the density of destinations in the residential neighborhood was positively associated with overall walking for transportation in the whole sample, with walking to work, and with walking to shops, while the density of destinations in the workplace neighborhood was positively associated with overall walking for transportation among workers.

In our study, the walkability of neighborhoods was assessed by considering the street network connectivity and the density of destinations. Our findings suggest that variations in neighborhood walkability also strongly influence walking for transportation in the French metropolitan region of Paris, as they do in the United States [44]. Our work also shows that these walkability effects were captured by the density of services rather than by the connectivity of the street network. Finally, it is particularly interesting to document that among workers, the density of destinations around the residence was not associated with overall walking for transport, but that the density of destinations around the workplace was, likely because most people are at work during daytime when services are open and because workers take opportunity of services around their workplace when available.

The distance to the primary supermarket was negatively related to walking to shops, but no such relationship was documented for walking to work. This finding corroborates studies that found that active commuting is less sensitive to distance in work trips than for other trip purposes [45], [46]. Increased walking to shops was found when the distance to the primary supermarket was inferior to 1000 m, which is coherent with the idea that 1000 m correspond to a 10 to 15 minute walk in an urban setting and encompass the easily accessible resources [47]. However, the relationship with the distance to the supermarket disappeared when the density of destinations was entered in the model, suggesting that the presence of a number of close shops rather than only the participant's primary supermarket determined the time spent in walking to shops.

None of the variables calculated along the itinerary to work or along the itinerary to the supermarket was associated with walking. A reason of this may be that the itineraries to work and to the supermarket that were considered were not the actual itineraries followed by the participants, but automatically determined street network itineraries. The assessment of actual itineraries in our ongoing RECORD GPS Study [48] will be useful to address this concern.

### Strengths and limitations

Strengths of the study include the complementary outcome variables considered to assess walking for transport and the definition of environmental variables not only around the residence but also around the workplace and around the itineraries to work and to one's supermarket. Strengths of this study also include the correspondence that we could establish between the exposures and the outcomes (e.g., shortest street network itinerary to work and self-reported walking to work).

However, a limitation is that the RECORD sample is not strictly representative of the Paris Ile-de-France population from which it was drawn, even if we a priori selected a panel of municipalities from the region to ensure the presence of people from all socioeconomic backgrounds (neighborhood-related selective participation in the sample has been investigated elsewhere [49]). Furthermore, the existence of selective residential migration processes did not allow us to demonstrate that the relationships between specific environmental factors and walking for transportation were attributable to a causal effect of these environments on walking [17]. Finally, sources of imprecision in the present study included: the fact that the actual itineraries to the workplace and supermarket were unknown (shortest network itineraries were used instead); the fact that contextual variables related to the participant's primary supermarket were examined in relation to walking to all shops and not only to the supermarket; and the use of self-reported rather than pedometer or accelerometry data for walking (even if the latter do not allow one to distinguish between the different forms of walking, e.g., for recreation or transport). Our questionnaire on walking was inspired from the short form of the International Physical Activity Questionnaire, but was not validated in its present form. However, we argue that the list of types of destinations that was provided helped the participants to remember their walking episodes.

## Conclusion

In conclusion, the density of services and the educational level of the residents were positively associated overall walking for transportation and walking to work and to one's primary supermarket. Residential neighborhood variables showed strong associations with walking, but there was some evidence that the assessment of these variables around the destinations of the trips contributed to improve the models. In particular, the environmental conditions around the workplace may notably influence the walking behavior of workers. Of potential interest for policymakers, our study shows that socially advantaged environments with a high density of destinations strongly promote walking for transportation.

## Supporting Information

Table S1 **Associations between individual characteristics and walking for transportation, the RECORD Study, 2007–2008.**
(DOCX)

Table S2 **Associations between environmental characteristics and walking for transportation, the RECORD Study, 2007–2008.** Data represent the associations between environmental factors not adjusted for each other and overall walking for transportation.
(DOCX)

Table S3 **Associations between individual characteristics and walking to work or walking to shops, the RECORD Study, 2007–2008.**
(DOCX)

Table S4 **Associations between distance to destinations and walking for transportation, the RECORD Study, 2007–2008.**
(DOCX)

Table S5 **Associations between each environmental factor and walking to work and walking to shops, the RECORD Study, 2007–2008.** Data represent associations between contextual factors not adjusted for each other and walking to work or shops. These tables show the estimated odds ratios represented with a confidence interval of 95%.
(DOCX)
