# Supplementary material for: Environmental Conditions around Itineraries to Destinations as Correlates of Walking for Transportation among Adults: The RECORD Cohort Study
Source: PLoS One. 2014 May 14;9(5):e88929. doi: 10.1371/journal.pone.0088929 (PMC4020748; doi:10.1371/journal.pone.0088929)
Supplement: Table S1 — Associations between individual characteristics and walking for transportation, the RECORD Study, 2007–2008. (DOCX) [file pone.0088929.s001.docx]

**Table S1** **Associations between individual characteristics and walking for transportation, the RECORD Study, 2007**–**2008**

| **Variables** | **Overall walking for transportation (n = 7105)**  **OR (95% CI)** | **Overall walking for transportation among workers (n = 4127)**  **OR (95% CI)** |
| --- | --- | --- |
| Men (vs. women) | 0.89 (0.81 – 0.98) | 0.88 (0.88 – 1.00) |
| Age (vs. 30-44) |  |  |
| 45–59 | 1.18 (1.07 – 1.30) | 1.15 (1.02 – 1.29) |
| 60–79 | 1.23 (1.09 – 1.39) | 1.13 (0.92 – 1.38) |
| Living alone (vs. as a couple) | 1.15 (1.04 – 1.26) | 1.20 (1.05 – 1.36) |
| Individual education (vs. no education) |  |  |
| Medium-low education | 1.69 (1.41 – 2.01) | 2.03 (1.60 – 2.57) |
| Medium-high education | 2.02 (1.69 – 2.42) | 2.25 (1.77 – 2.86) |
| High education | 2.05 (1.71 – 2.47) | 2.43 (1.89 – 3.12) |
| Occupation (vs. blue collar workers) |  |  |
| Low white-collar workers | 1.19 (1.04 – 1.36) | 1.09 (0.90 – 1.32) |
| Intermediate occupations | 1.10 (0.88 – 1.37) | 1.03 (0.78 – 1.36) |
| High white-collar workers | 1.00 (0.86 – 1.16) | 0.92 (0.73 – 1.14) |
| Perceived financial strain | 1.00 (0.88 – 1.15) | 1.13 (0.95 – 1.34) |
| Household income (vs. low income) |  |  |
| Medium-low income | 1.08 (0.95 – 1.23) | 0.92 (0.78 – 1.10) |
| Medium-high income | 1.05 (0.91 – 1.21) | 0.92 (0.76 – 1.11) |
| High income | 1.04 (0.90 – 1.20) | 0.94 (0.77 – 1.15) |
| Homeownership (vs. not) | 1.06 (0.96 – 1.17) | 1.08 (0.95 – 1.23) |
| Human Development Index of country of birth (vs. France) |  |  |
| Low | 0.81 (0.66 – 1.01) | 0.96 (0.74 – 1.25) |
| Medium | 1.08 (0.95 – 1.23) | 1.12 (0.94 – 1.32) |
| High (other than France) | 0.99 (0.85 – 1.15) | 1.19 (0.98 – 1.44) |
