# Supplementary material for: Environmental Conditions around Itineraries to Destinations as Correlates of Walking for Transportation among Adults: The RECORD Cohort Study
Source: PLoS One. 2014 May 14;9(5):e88929. doi: 10.1371/journal.pone.0088929 (PMC4020748; doi:10.1371/journal.pone.0088929)
Supplement: Table S2 — Associations between environmental characteristics and walking for transportation, the RECORD Study, 2007–2008. Data represent the associations between environmental factors not adjusted for each other and overall walking for transportation. (DOCX) [file pone.0088929.s002.docx]

**Table S2** **Associations between environmental characteristics and walking for transportation, the RECORD Study, 2007-2008**

| **Variables** | **Overall walking for transportation (n = 7105)** | **Overall walking for transportation among workers (n = 4127)** |
| --- | --- | --- |
|  | **OR (95% CI)*** | **OR (95% CI)*** |
| Residential neighborhood education (vs. low) |  |  |
| Mid-low | 1.21 (1.07 - 1.37) | 1.11 (0.94 - 1.31) |
| Mid-high | 1.43 (1.26 - 1.63) | 1.40 (1.18 - 1.67) |
| High | 1.34 (1.18 - 1.54) | 1.32 (1.11 - 1.57) |
| Density of destinations around the residence (vs. low) |  |  |
| Mid-low | 1.31 (1.16 - 1.47) | 1.12 (0.96 - 1.31) |
| Mid-high | 1.64 (1.46 - 1.86) | 1.29 (1.10 - 1.50) |
| High | 2.20 (1.94 - 2.49) | 1.43 (1.22 - 1.68) |
| Proportion of parks around the residence (vs. low) |  |  |
| Mid-low | 1.18 (1.04 - 1.32) | 1.11 (0.95 - 1.30) |
| Mid-high | 1.25 (1.11 - 1.40) | 1.23 (1.05 - 1.44) |
| High | 0.98 (0.87 - 1.10) | 1.04 (0.89 - 1.22) |
| Workplace neighborhood education (vs. low) |  |  |
| Mid-low | – | 1.07 (0.91 - 1.25) |
| Mid-high | – | 1.10 (0.94 - 1.29) |
| High | – | 1.24 (1.06 - 1.46) |
| Density of destinations around the workplace (vs. low) |  |  |
| Mid-low | – | 1.06 (0.91 - 1.24) |
| Mid-high | – | 1.25 (1.07 - 1.46) |
| High | – | 1.43 (1.22 - 1.67) |
| Proportion of parks around the workplace (vs. low) |  |  |
| Mid-low | – | 1.12 (0.95 - 1.32) |
| Mid-high | – | 1.22 (1.04 - 1.43) |
| High | – | 0.99 (0.84 - 1.17) |

*Models adjusted for age, sex, marital status, individual education, occupation, home ownership status, perceived financial strain, household income and the level of human development of the country of birth
