# Supplementary material for: Environmental Conditions around Itineraries to Destinations as Correlates of Walking for Transportation among Adults: The RECORD Cohort Study
Source: PLoS One. 2014 May 14;9(5):e88929. doi: 10.1371/journal.pone.0088929 (PMC4020748; doi:10.1371/journal.pone.0088929)
Supplement: Table S3 — Associations between individual characteristics and walking to work or walking to shops, the RECORD Study, 2007–2008. (DOCX) [file pone.0088929.s003.docx]

**Table S3** **Associations between individual characteristics and walking to work or walking to shops, the RECORD Study, 2007**–**2008**

| **Variables** | **Walking to work (n = 4127)**  **OR (95% CI)** | **Walking to shops (n=6958)**  **OR (95% CI)** |
| --- | --- | --- |
| Men (vs. women) | 0.94 (0.83 – 1.08) | 0.74 (0.67 – 0.81) |
| Age (vs. 30-44) |  |  |
| 45–59 | 1.01 (0.89 – 1.13) | 1.25 (1.13 – 1.38) |
| 60–79 | 0.39 (0.31 – 0.49) | 2.49 (2.20 – 2.82) |
| Living alone (vs. as a couple) | 1.00 (0.87 – 1.13) | 1.07 (0.97 – 1.19) |
| Individual education (vs. no education) |  |  |
| Medium-low education | 1.44 (1.13 – 1.82) | 1.43 (1.20 – 1.72) |
| Medium-high education | 1.46 (1.15 – 1.86) | 1.64 (1.36 – 1.97) |
| High education | 1.42 (1.11 – 1.83) | 1.62 (1.34 – 1.96) |
| Occupation (vs. blue collar workers) |  |  |
| Low white-collar workers | 0.90 (0.74 – 1.09) | 1.05 (0.92 – 1.21) |
| Intermediate occupations | 1.34 (1.02 – 1.76) | 0.88 (0.70 – 1.09) |
| High white-collar workers | 1.15 (0.92 – 1.43) | 0.85 (0.73 – 1.00) |
| Perceived financial strain | 0.95 (0.80 – 1.12) | 1.02 (0.89 – 1.17) |
| Household income (vs. low income) |  |  |
| Medium-low income | 1.08 (0.91 – 1.29) | 0.97 (0.85 – 1.11) |
| Medium-high income | 1.11 (0.92 – 1.35) | 0.96 (0.83 – 1.11) |
| High income | 1.08 (0.89 – 1.32) | 0.93 (0.80 – 1.08) |
| Homeownership (vs. not) | 1.14 (1.01 – 1.30) | 1.03 (0.93 – 1.14) |
| Human Development Index of country of birth |  |  |
| Low | 1.46 (1.14 – 1.89) | 0.73 (0.59 – 0.91) |
| Medium | 1.43 (1.21 – 1.68) | 1.01 (0.88 – 1.15) |
| High (other than France) | 1.49 (1.23 – 1.81) | 0.81 (0.69 – 0.94) |
