# Supplementary material for: Environmental Conditions around Itineraries to Destinations as Correlates of Walking for Transportation among Adults: The RECORD Cohort Study
Source: PLoS One. 2014 May 14;9(5):e88929. doi: 10.1371/journal.pone.0088929 (PMC4020748; doi:10.1371/journal.pone.0088929)
Supplement: Table S4 — Associations between distance to destinations and walking for transportation, the RECORD Study, 2007–2008. (DOCX) [file pone.0088929.s004.docx]

| **Table S4 Associations between distance to destinations and walking for transportation, the RECORD Study, 2007**–**2008*** | | | |
| --- | --- | --- | --- |
| **Variables** | **Overall walking for transportation among workers (n = 4127)**  **OR (95% CI)** | **Walking to work (n = 4127)**  **OR (95% CI)** | **Walking to shops (n = 6958)**  **OR (95%CI)** |
| Distance between the residence and the workplace (vs. >5 km) |  |  |  |
| 1 km – 5 km | 1.01 (0.89 – 1.15) | 0.99 (0.87 – 1.13) | – |
| <1 km | 0.95 (0.73 – 1.24) | 0.83 (0.64 – 1.09) | – |
| Distance between the residence and the supermarket |  |  |  |
| 1 km – 5 km | – | – | 0.99 (0.85 – 1.16) |
| <1 km | – | – | 1.23 (1.06 – 1.43) |

*Models adjusted for age, sex, marital status, individual education, occupation, home ownership status, perceived financial strain, household income, and the level of human development of the country of birth
