# Supplementary material for: Environmental Conditions around Itineraries to Destinations as Correlates of Walking for Transportation among Adults: The RECORD Cohort Study
Source: PLoS One. 2014 May 14;9(5):e88929. doi: 10.1371/journal.pone.0088929 (PMC4020748; doi:10.1371/journal.pone.0088929)
Supplement: Table S5 — Associations between each environmental factor and walking to work and walking to shops, the RECORD Study, 2007–2008. Data represent associations between contextual factors not adjusted for each other and walking to work or shops. These tables show the estimated odds ratios represented with a confidence interval of 95%. (DOCX) [file pone.0088929.s005.docx]

**Table S5** **Associations between each environmental factor and walking to work and walking to shops, the RECORD Study, 2007-2008**

| **Variables** | **Walking to work (n = 4127)** | **Walking to shops (n = 6958)** |
| --- | --- | --- |
|  | **OR (95% CI)*** | **OR (95% CI)**** |
| Residential neighborhood education (vs. low) |  |  |
| Mid-low | – | 1.12 (0.98 - 1.27) |
| Mid-high | – | 1.32 (1.16 – 1.51) |
| High | – | 1.27 (1.11 - 1.46) |
| Density of destinations around the residence (vs. low) |  |  |
| Mid-low | 1.21 (1.03 – 1.42) | 1.17 (1.04 - 1.33) |
| Mid-high | 1.53 (1.31 – 1.80) | 1.53 (1.35 - 1.74) |
| High | 1.75 (1.50 – 2.05) | 1.69 (1.48 - 1.91) |
| Proportion of parks around the residence (vs. low) |  |  |
| Mid-low | – | 1.08 (0.95 - 1.22) |
| Mid-high | – | 1.14 (1.01 - 1.29) |
| High | – | 1.00 (0.88 - 1.13) |
| Workplace neighborhood education (vs. low) |  |  |
| Mid-low | 1.28 (1.10 – 1.50) | – |
| Mid-high | 1.20 (1.02 – 1.41) | – |
| High | 1.62 (1.38 – 1.90) | – |
| Density of destinations around the workplace (vs. low) |  |  |
| Mid-low | 1.19 (1.02 – 1.40) | – |
| Mid-high | 1.49 (1.27 – 1.74) | – |
| High | 1.71 (1.46 – 2.00) | – |
| Proportion of parks around the workplace (vs. low) |  |  |
| Mid-low | 1.39 (1.18 – 1.64) | – |
| Mid-high | 1.35 (1.15 – 1.59) | – |
| High | 1.25 (1.06 – 1.49) | – |

*Models adjusted for age, sex, marital status, individual education, occupation, home ownership status, perceived financial strain, household income and the level of human development of the country of birth

** Model further adjusted for distance to the closest supermarket
